# Quantitative analysis of 11‐dehydrocorticosterone and corticosterone for preclinical studies by liquid chromatography/triple quadrupole mass spectrometry

**DOI:** 10.1002/rcm.8610

**Published:** 2020-02-11

**Authors:** Manu Verma, Karen Sooy, George Just, Mark Nixon, Ruth Morgan, Ruth Andrew, Karen E. Chapman, Natalie Z.M. Homer

**Affiliations:** ^1^ University/BHF Centre for Cardiovascular Sciences, Queen's Medical Research Institute, University of Edinburgh 47 Little France Crescent Edinburgh EH16 4TJ UK; ^2^ Mass Spectrometry Core, Edinburgh Clinical Research Facility, Queen's Medical Research Institute University of Edinburgh 47 Little France Crescent Edinburgh EH16 4TJ UK

## Abstract

**Rationale:**

The activity of the glucocorticoid activating enzyme 11β‐hydroxysteroid dehydrogenase type‐1 (11βHSD1) is altered in diseases such as obesity, inflammation and psychiatric disorders. In rodents 11βHSD1 converts inert 11‐dehydrocorticosterone (11‐DHC) into the active form, corticosterone (CORT). A sensitive, specific liquid chromatography/tandem mass spectrometry method was sought to simultaneously quantify total 11‐DHC and total and free CORT in murine plasma for simple assessment of 11βHSD1 activity in murine models.

**Methods:**

Mass spectrometry parameters were optimised and a method for the chromatographic separation of CORT and 11‐DHC was developed. Murine plasma was prepared by 10:1 chloroform liquid–liquid extraction (LLE) for analysis. Limits of quantitation (LOQs), linearity and other method criteria were assessed, according to bioanalytical method validation guidelines.

**Results:**

Reliable separation of 11‐DHC and CORT was achieved using an ACE Excel 2 C18‐AR (2.1 × 150 mm; 2 μm) fused core column at 25°C, with an acidified water/acetonitrile gradient over 10 min. Analytes were detected by multiple reaction monitoring after positive electrospray ionisation (*m/z* 345.1.1 ➔ 121.2, *m/z* 347.1 ➔ 121.1 for 11‐DHC and CORT, respectively). The LOQs were 0.25 and 0.20 ng/mL for 11‐DHC and CORT, respectively.

**Conclusions:**

This LC/MS method is suitable for the reliable analysis of 11‐DHC and CORT following simple LLE of murine plasma, bringing preclinical analysis in line with recommendations for clinical endocrinology and biochemistry.

## 1 INTRODUCTION

Glucocorticoids are essential for the regulation of metabolism, the stress response and inflammation. Glucocorticoid action is controlled at two levels: first by the hypothalamic–pituitary axis, a negative feedback loop which determines the levels of circulating glucocorticoids, and secondly by metabolism of glucocorticoids in the tissues. The best documented example of tissue metabolism of glucocorticoids is 11β‐hydroxysteroid dehydrogenase 1 (11βHSD1).^1^ In rodents this enzyme converts inert glucocorticoid 11‐dehydrocorticosterone (11‐DHC) into the active form, corticosterone (CORT).

The activity of 11βHSD1 is altered in several disease states including obesity, inflammation and psychiatric disorders.^2^ The importance of this enzyme has been revealed by murine models of global or tissue‐specific disruption^3^, ^4^ or over‐expression^5^ of the enzyme. Demonstrating the activity of this enzyme is essential in the validation of these murine models and in the understanding of the role of this enzyme in health and disease. The preclinical field is hampered by the lack of a robust assay for the enzyme substrate, 11‐dehydrocorticosterone (11‐DHC). Antibodies to 11‐DHC are not commercially available, and only rarely reported.^6^ Immunoassays for CORT have variable cross‐reactivity with other endogenous steroids, of which there are many. Similar problems in clinical biochemistry have been overcome by the use of tandem mass spectrometry (MS/MS); non‐selective immunoassays for sex steroids are now no longer acceptable for publication.^7^ Furthermore, the advantage of LC/MS analysis is the ability to analyse more than one compound in a sample. In 2005 Ronquist et al^8^ detailed a 16‐min LC/MS method that measured levels of CORT and 11‐DHC in murine liver and adipose; however, this was not applied to blood and used trifluoracetic acid as a modifier in the chromatographic method. A 6‐min on‐line extraction LC/MS method for CORT and 11‐DHC analysis has been described and applied to human and rat placenta,^9^ but again it has not been applied to blood. Peti et al reported an 11‐min LC/MS method for CORT, 11‐DHC, progesterone, aldosterone, cortisol and cortisone analysis using a high‐resolution TripleTOF 5600 mass spectrometer,^10^ where the limit of quantitation (LOQ) of CORT and 11‐DHC was 3.9 ng/mL. Li et al developed a 6‐min LC/MS method for CORT analysis (LOQ of 1 ng/mL), but not 11‐DHC, in mouse plasma.^11^ A recent human clinical study by Taylor et al^12^ describes a 19.7‐min‐long LC/MS method for a panel of 13 steroids in serum. The method includes CORT, with an LOQ of 0.25 ng/mL, but 11‐DHC is not included in this panel. A 14‐min steroid profiling human plasma method includes CORT with an LOQ of 0.5 ng/mL, but not 11‐DHC.^13^ To date there has not been a validated method focusing only on CORT and 11‐DHC in murine plasma.

In this study we have used a murine model of inflammation known to increase 11βHSD1 activity^14^ to report a validated, sensitive liquid chromatography/tandem mass spectrometry (LC/MS/MS)‐based method for quantifying plasma levels of the substrate as well as the product of 11βHSD1, 11‐DHC and CORT. This method for CORT analysis was sensitive enough to analyse the “free” CORT levels in murine plasma, i.e. the circulating steroid unbound to corticosteroid binding globulin (CBG), which is thought to be the biologically active portion of CORT.

## 2 EXPERIMENTAL

### 2.1 Chemicals, reagents and consumables

HPLC‐grade chloroform was from Rathburn Chemicals (Walkerburn, UK). LC/MS grade dichloromethane, isopropanol, methanol, acetonitrile and water were from Fisher Scientific (Loughborough, UK). Formic acid (FA) was from Sigma Aldrich (Gillingham, UK), [9,11,12,12‐^2^H_4_]‐Cortisol (d4F) was from CDN (Pointe‐Claire, Canada) and the steroids, CORT, 11‐DHC, and epi‐corticosterone (epi‐CORT), were from Steraloids (Newport, RI, USA).

### 2.2 Stock solutions and calibration standards

Stock solutions (1 mg/mL) of analytes (CORT, 11‐DHC) and internal standard (d4‐cortisol (d4F) and epi‐CORT), in methanol, were stored at −20°C and were further diluted in methanol on the day of use. Calibration standards were diluted in methanol from 0.01 to 500 ng/mL on the day of preparation.

### 2.3 Chromatographic and mass spectrometric conditions

Analysis was performed using an Acquity Classic ultra‐performance liquid chromatography (UPLC) system (Waters, Wilmslow, UK) interfaced to a QTRAP 5500 mass spectrometer (AB Sciex, Warrington, UK), operated using Sciex Analyst® 1.5.1 software. Data was processed for quantitation using MultiQuant™ software (Sciex; version 3.0.2). Samples were injected (30 μL) onto the UPLC system using a partial loop with needle overfill (PLNO) on a 50‐μL loop. Chromatographic separation was achieved using an ACE Excel 2 C18‐AR LC column (2.1 × 150 mm; 2 μm; Advanced Chromatography Technologies, Aberdeen, UK) protected by a KrudKatcher™ Ultra (Phenomenex, Macclesfield, UK) as in‐line filter. The mobile phase was water (0.1% FA; v/v) and acetonitrile (0.1% FA, v/v). Gradient elution was performed from 30 to 90% acetonitrile with a run time of 10 min (Table 1). The mass spectrometer was operated in positive ion electrospray ionisation (ESI) mode using a TurboIonSpray source and data collected in unit resolution (0.7 *m/z* units full width at half maximum). The TurboIonSpray source was operated at 550°C with an IonSpray voltage of 5 kV, a curtain gas pressure of 20 psi, and nitrogen nebuliser ion source gas 1 (GS1) and heater ion source gas 2 (GS2) pressures of 40 psi and 60 psi, respectively. Compound‐specific parameters were optimised for selected reaction monitoring (SRM) transitions (Table 1) by infusing 100 ng/mL of each steroid standard solution into the source at 3 μL/min with a collision‐activated dissociation (CAD) gas at the medium setting of 2.6 × 10^−5^ Torr. The curtain, source, exhaust and CAD gases were delivered using an MS Table 1N nitrogen table (Peak Scientific, Inchinnan, UK).

**Table 1:** Instrument settings for CORT, 11‐DHC and internal standards

Chromatographic conditions (flow rate 0.5 mL/min)		
Time (min)	Mobile phase A (%): water (0.1% FA, v/v)	Mobile phase B (%): acetonitrile (0.1% FA, v/v)
0	70	30
1.5	70	30
3	10	90
4	10	90
6	70	30
10	70	30

Chromatographic conditions (flow rate 0.5 mL/min)						
Analyte	Mass transition (*m/z*)	Time (ms)	DP	EP	CE	CXP
11‐DHC	345.1 → 121.2 (345.1 → 90.9)	30	51 61	10 10	33 71	8 10
CORT and epi‐CORT	347.1 → 121.1 (347.1 → 91.1)	30	66 66	10 10	69 69	8 8
d4F	367.1 → 121.1 (367.0 → 91.1)	30	121 121	10 10	25 25	20 20

1 Conditions established following electrospray ionisation at 5 kV, 550°C. FA = formic acid; DP = declustering potential (V); CE = collision energy (eV); CXP = collision cell exit potential (V).

Quantitative ion with qualitative ion shown in brackets.

### 2.4 Sample collection and preparation

#### 2.4.1 Preclinical murine model

Male C57BL6 mice (>10 weeks old; Harlan Olac, Bicester, UK) were housed 4 to 5 per cage under standard conditions on a 12 h light/dark cycle (lights on at 7:00 am) with *ad libitum* access to food (standard chow) and water. All procedures were performed under the aegis of the UK Animals (Scientific Procedures) Act, 1986, and the EU directive 2010/63/EU and with local ethical committee approval.

#### 2.4.2 Administration of lipopolysaccharide (LPS) by intra‐peritoneal (ip) injection

Mice were administered either 0.9% saline solution (vehicle) or LPS (100 μg/kg; derived from *Escherichia coli* 0111:B4 (Sigma‐Aldrich)) by a single ip injection between 07:30 h and 09:15 h. Mice were culled by decapitation 3 h later.

#### 2.4.3 Administration of LPS intra‐nasally

Mice were administered LPS (1 mg/mL) intra‐nasally at 09:00 h. A control group was left untreated. Mice were culled by decapitation 24 h later and blood collected for steroid analysis.

#### 2.4.4 Blood collection

Trunk blood from the mice was collected into EDTA‐coated tubes, centrifuged (1000 *g*, 10 min, 4°C) and the plasma transferred to labelled vials and stored at −80°C prior to steroid analysis.

### 2.5 Sample extraction protocol

#### 2.5.1 Steroid extraction from murine plasma by liquid–liquid extraction (LLE)

Murine plasma (50 or 150 μL) was aliquoted into glass tubes, enriched with internal standards d4F and epi‐CORT (2.5 ng each) and extracted by adding chloroform (0.5 or 1.5 mL) and mixing well. After vortexing, the supernatant was transferred to a clean 4.5‐mL glass tube (75 × 10 mm), reduced to dryness under oxygen‐free nitrogen (60°C), reconstituted in water/acetonitrile (30:70, v/v; 70 μL) and transferred to an autosampler vial.

#### 2.5.2 Preparation of plasma for analysis of free CORT

Plasma (150 μL) was incubated (37°C; 30 min) before being applied to an Ultracel‐30 membrane in an Amicon ultra‐centrifugal filter unit (Millipore, Livingstone, UK) and subjected to centrifugation (14,000 *g*, 37°C; 30 min). The ultrafiltrate (150 μL) was subjected to 10:1 chloroform steroid extraction as described in section 2.5.1, and the extract was assessed for CORT levels which represents the free component.

### 2.6 Method validation

To validate the developed LLE LC/MS/MS assay, the recovery, linearity and lower limits of detection and quantitation were determined in accordance with the European Medicines Agency bioanalytical method validation guidelines.^15^, ^16^

#### 2.6.1 Recovery of 11‐DHC and CORT from water

The absolute recovery was assessed by enriching water with 2.5 ng of 11‐DHC and CORT and using the LLE method described (section 2.5.1). The peak areas of CORT and 11‐DHC in these enriched aqueous samples were compared with those from pure water subjected to the extraction procedure and post‐spiked with CORT and 11‐DHC (2.5 ng).

#### 2.6.2 Recovery of internal standards, epi‐CORT and d4F, from plasma

Recoveries of d4F and epi‐CORT from pooled control murine plasma (150 μL) enriched with d4F and epi‐CORT (2.5 ng) were ascertained using the extraction procedure described in sections 2.5.1 and 2.5.2. The peak areas of d4F and epi‐CORT from these ‘pre‐spiked’ plasma samples were divided by those of plasma that had been post‐spiked with d4F and epi‐CORT (2.5 ng) and the percentage recovery of the internal standards from mouse plasma was calculated. The pre‐spiked values were compared with those from pure solutions of d4F and epi‐CORT (2.5 ng) to calculate the matrix effects on the internal standards.

#### 2.6.3 Assay specificity

The ion ratio of quantifier and qualifier ions for each analyte was assessed in all biological samples. The criterion for acceptance is a ratio within 20% of the ratio for analytical standards, as defined in bioanalytical validation guidelines.^15^, ^16^

#### 2.6.4 Sensitivity and linearity (LOD, LOQ, accuracy and precision)

Six sets of calibration standards (0.1–400 ng) were prepared and extracted by LLE and analysed by LC/MS/MS. The limits of detection and quantitation (LOD and LOQ, respectively) were calculated using the standard deviation of the response (σ) and the slope (m) using the following equations: LOD = 3.3(σ/m) and LOQ = 10(σ/m).^16^ Linearity was evaluated in calibration standards generated on six different days, assessing the peak area ratios of the analyte (CORT and 11‐DHC) divided by that of the internal standard (d4F and epi‐CORT). Regression analyses with weighting options (equal, 1/x, 1/x^2^) were explored. The results derived using the two internal standards were compared.

#### 2.6.5 Reproducibility

The accuracy and precision were determined by assessing calibration standards, following extraction, at the LLOQ and at low, medium and high amounts of 11‐DHC and CORT (2.5, 10, 150 ng) in replicates of six prepared on the same day (intra) and of three prepared on different days (inter). The precision was calculated as the relative standard deviation of the mean (RSD), calculated as the standard deviation of the replicates divided by the average of the replicates and multiplied by 100. The amount of steroid in each sample was calculated using the calibration curve and accuracy calculated as the relative mean error (RME), where the calculated amount minus the theoretical amount is divided by the theoretical amount multiplied by 100. According to bioanalytical guidelines, the accuracy and precision are considered acceptable when <15% for all points and <20% for the LOQ.

#### 2.6.6 Stability

Extracts of three aliquots of plasma, enriched with d4F and epi‐CORT (2.5 ng), were analysed immediately after preparation and then allowed to remain at 10°C in the autosampler for 12 h, or at −20°C for 7 days or 2 months prior to repeat analysis. The concentration of 11‐DHC and CORT was calculated from the standard curve prepared and stored with the samples and calculated as a percentage of the initial value. The stability was acceptable if the change in concentration measured was <20%.

### 2.7 Method application

The amounts of total and free CORT and total 11‐DHC were quantified in murine plasma. The concentrations of endogenous steroids were compared before and after LPS treatment in two separate mouse experiments; intra‐peritoneal injection and intra‐nasal injection in C57BL6 mice.

## 3 RESULTS AND DISCUSSION

### 3.1 Mass spectrometric parameters

All steroidal analytes and internal standards generated singly charged positive ions and underwent transitions in multiple reaction monitoring (MRM) mode to product ions typical of pregnene steroids. The common product ion of *m/z* 121 is from the A‐ring of the steroid structure, first described by Williams et al^17^ using stable isotope labelled testosterone to identify fragments from collision‐induced dissociation. It was also shown by Ronquist‐Nii et al in corticosteroids.^8^

### 3.2 Chromatographic development

**Figure 1:**
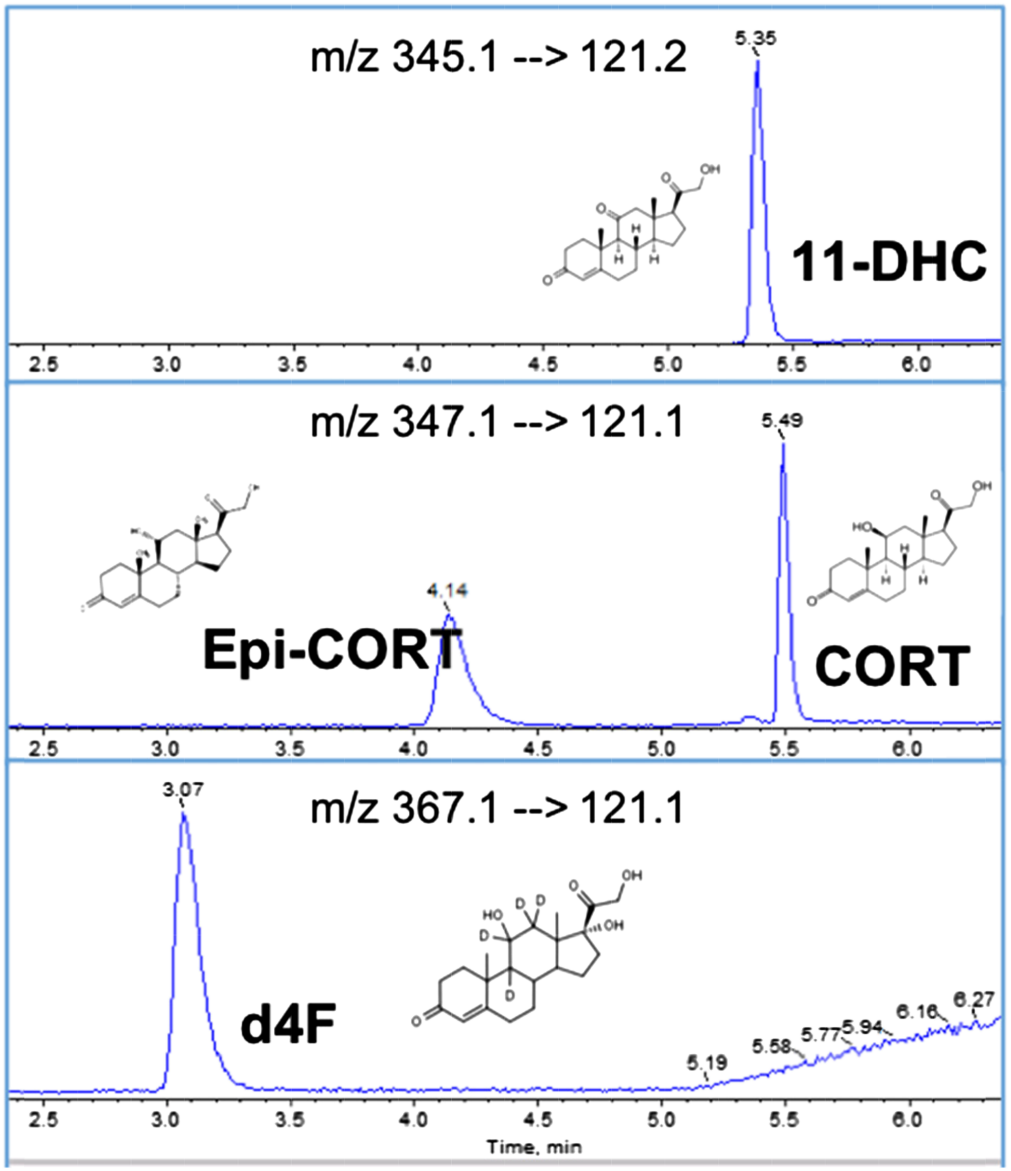
Extracted ion chromatograms of 11‐DHC, CORT, epi‐CORT and d4F demonstrating chromatographic resolution of isobaric epimers CORT and epi‐CORT and of 11‐DHC and CORT

Due to the mass difference in molecular mass between 11‐DHC and CORT of only 2 Da, the natural ^2^H and ^13^C isotopologues of 11‐DHC register signals within the corticosterone MRM transition. Thus, chromatographic separation of 11‐DHC and CORT was essential. Initially, a Sunfire C18 column (100 × 2.1 mm; 3.5 μm; Waters) was trialled for separation of the steroids, but selectivity was improved using an ACE Excel C18‐AR column (150 × 2.1 mm; 2 μm; ACT, Aberdeen, UK), due to the longer column length, smaller particle size and a modified stationary phase. This not only afforded improved sensitivity but also robust, reliable chromatographic separation. Under these conditions, the isobaric internal standard, epi‐CORT, was resolved temporally (Figure 1), which was also necessary.

### 3.3 Method validation

#### 3.3.1 Recovery following LLE of 150 μL murine plasma

Protein precipitation was pursued initially, but yielded considerable chromatographic interference around the retention times of epi‐CORT, CORT and 11‐DHC. Liquid–liquid extraction (LLE) using chloroform (10:1) was assessed and gave a much cleaner extract with no interfering peaks. The results obtained following extraction of two different volumes of pooled mouse plasma (50 and 150 μL) were compared. The variability in the calculated amounts at 50 μL (32.1% for CORT and 38.3% for 11‐DHC) was much larger than that seen in the 150‐μL extracts (13.4% for CORT and 14.2% for 11‐DHC). Therefore, 150 μL was considered the acceptable and reliable volume that would still allow data to be collected from individual experimental animals. Using the 10:1 chloroform LLE approach, average and acceptable recoveries of 89.1% 11‐DHC and 87.2% of CORT from aqueous solution were obtained, calculated by comparing the peak areas of 11‐DHC and CORT pre‐spiked with that of an extract that had been post‐spiked with 11‐DHC and CORT (Table 2). The recovery from plasma (*n* = 3; 150 μL) of the non‐endogenous internal standards epi‐CORT (89 ± 2.9%) and d4F (93.1 ± 2.2%) was excellent.

**Table 2:** Recovery and indices of intra‐ and inter‐day precision and accuracy for quantitation of mouse plasma enriched at LOQ (0.25 and 0.20 ng/mL), low (2.5), medium (10) and high (150) levels of 11‐DHC and CORT

Amount (ng/mL)	% recovery	Intra‐day		Inter‐day	
	Mean ± RSD	Precision, RSD (%)	Accuracy, RME (%)	Precision, RSD (%)	Accuracy, RME (%)
0.25 (11‐DHC) 0.20 (CORT)	87.2 ± 3.4 89.1 ± 2.0	10.13 9.80	15.32 17.30	12.92 18.37	12.39 10.92
2.5 (11‐DHC) 2.5 (CORT)	89.5 ± 2.1 86.1 ± 3.1	4.19 4.70	3.94 8.11	14.74 11.21	10.27 8.45
10 (11‐DHC) 10 (CORT)	90.2 ± 5.4 86.3 ± 2.4	4.31 5.22	8.23 7.32	3.97 4.23	10.20 2.50
150 (11‐DHC) 150 (CORT)	89.5 ± 3.4 87.3 ± 2.4	6.23 7.90	1.20 2.50	9.44 12.30	5.56 3.50

RSD = relative standard deviation; RME = relative mean error.

#### 3.3.2 Assay specificity for LLE

No interfering peaks were seen in chromatographic traces of LLE‐extracted plasma for the internal standards d4F or epi‐CORT. The quantifier/qualifier ratio of 11‐DHC and CORT in six different plasma extracts was 0.72 ± 0.04 and 0.79 ± 0.06, respectively, in close agreement with that of extracted analytical standards (0.75 ± 0.02 for 11‐DHC and 0.81 ± 0.04 for CORT).

#### 3.3.3 Sensitivity (LOD and LOQ) and linearity

The LOD and LOQ were calculated by extrapolation to be 0.1 ng/mL and 0.25 ng/mL for 11‐DHC and 0.10 ng and 0.20 ng/mL for CORT. Based on regression parameters, d4F was consistently found to be the best internal standard for 11‐DHC while epi‐CORT was the best internal standard for CORT. This was determined by assessing the widest dynamic range and the best line fit for the calibration curves. The calibration curves were linear over the range 0.1–500 ng/mL for 11‐DHC and CORT. The mean regression coefficients of the standard curves (*n* = 6) were r^2^ = 0.995 ± 0.003 for 11‐DHC and r^2^ = 0.997 ± 0.002 for CORT, with weighting of 1/x applied for optimal fitting of the lowest amounts. This is an improvement on previously reported analysis of CORT in murine plasma by tandem mass spectrometry (LOQ of 1 ng/mL^11^). Of importance, their method did not detect or report the amount of 11‐DHC in the murine plasma.

#### 3.3.4 Accuracy, precision, reproducibility

The precision and accuracy were acceptable for 11‐DHC and CORT at low, medium and high levels (2.5, 10 and 150 ng/mL) (Table 2). When applying the LOQs to a volume of 150 μL the levels of 11‐DHC and CORT fell comfortably above the LOQ (0.25 ng/mL for 11‐DHC and 0.20 ng/mL for CORT) and the upper limit of the assay (500 ng for 11‐DHC and CORT).

#### 3.3.5 Sample stability

The stability of the extracts was acceptable upon short‐term storage, changing to 97.2% for CORT and 96.4% for 11‐DHC (autosampler at 10°C for 12 h). Storage at −20°C for 1 week saw a change to 95.4% and 94.2% for 11‐DHC, and, longer term (2 months at −20°C), this changed to 93.1% for CORT and 90.8% for 11‐DHC. These assessments reflect conditions of normal laboratory practice and are acceptable.

### 3.4 Method application

The plasma from 53 individual mice, treated with LPS or vehicle (0.9% saline) (sections 2.4.1 and 2.4.2), were analysed using the validated protocol, where CORT was found to range between 52 and 480 ng/mL (150–1387 nM) and 11‐DHC was detected in the range 3–40 ng/mL (4–120 nM), comfortably within the validated ranges of the assay (0.57–1445 nM for CORT, 0.7–1453 nM for 11‐DHC).

**Figure 2:**
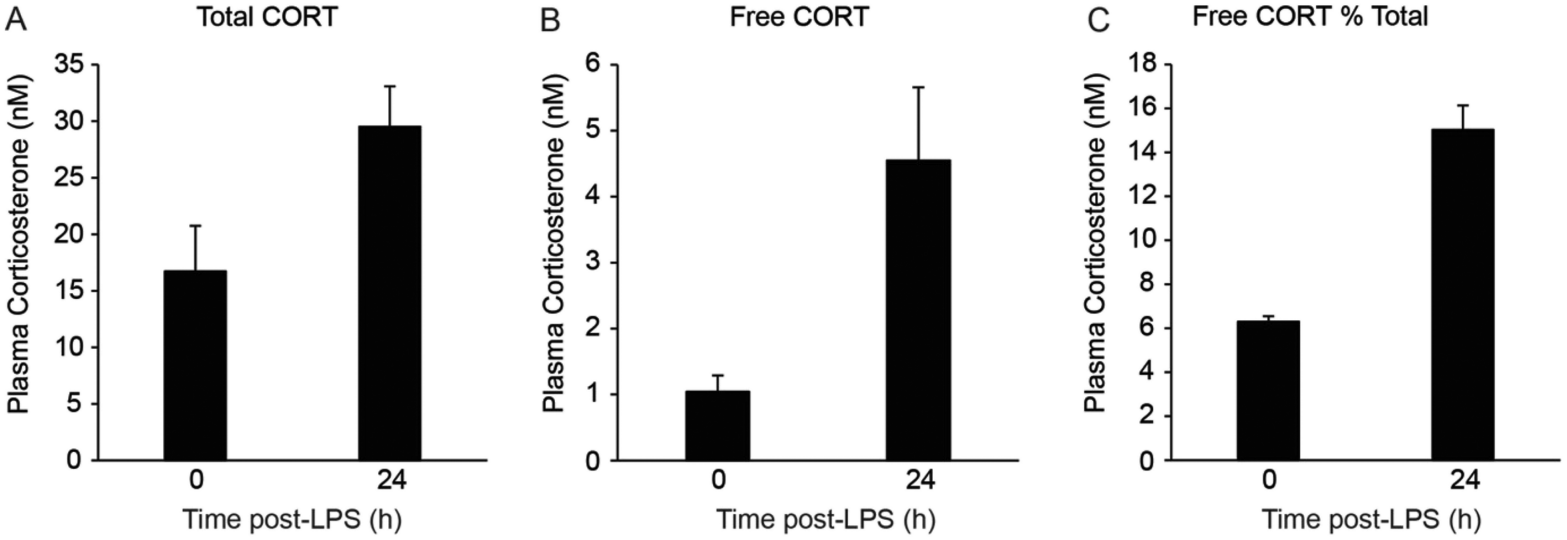
Concentrations of corticosterone (CORT) in mouse plasma, 3 h following ip injection of vehicle (0.9% saline) or 100 μg/kg lipopolysaccharide (LPS). Measured amounts were within the validated range. Data are mean ± SEM (*n* = 7/group)

Furthermore, the extraction protocol was applied to assess total and free CORT in 14 mouse plasma samples (section 2.4.3). The plasma was subjected to ultrafiltration, the filtrate was extracted by LLE (section 2.5.2) and analysed following the validated method, with samples falling in the range 1–7 nM CORT, within the validation of the method. Treatment of the mice with LPS sees an increase not only in total CORT, but also in free CORT. The concentration of the available pool of CORT indicates that free steroids increased from 6 to 15% of the total CORT, following LPS treatment (Figure 2).

## 4 CONCLUSIONS

The use of immunoassays in steroid biochemistry is gradually being replaced by chromatographic/mass spectrometric methods in the clinical research arena but this presents additional challenges in the preclinical field due to limited sample volumes from small animals. Nonetheless, advancing technology is now bringing these within reach allowing improvements in the specificity of biochemical data.

The LC/MS/MS method presented here allows reliable analysis of active and inactive glucocorticoids in plasma from individual animals. In contrast to previous methods of plasma steroid extraction, some of which used more costly solid‐phase extraction (SPE), the present method uses a relatively simple and cost‐effective liquid–liquid extraction (LLE) with highly efficient recoveries of the steroid analytes (~90%). It is likely that other steroids will be present within the plasma extract, offering possibilities of broader spectrum data for individual animals, depending upon the research question. Under current conditions, this approach consumed 150 μL of plasma, but with technological advances already available beyond the instrumental specifications described here it will probably lead to gains in this field of preclinical research.
